# Supplementary material for: The complete methylome of Helicobacter pylori UM032
Source: BMC Genomics. 2015 Jun 2;16(1):424. doi: 10.1186/s12864-015-1585-2 (PMC4450513; doi:10.1186/s12864-015-1585-2)

The complete methylome of *Helicobacter pylori* UM032

Woon Ching Lee[1], Brian P. Anton[2], Susana Wang[3], Primo Baybayan[3], Siddarth Singh[4], Meredith Ashby[3], Eng Guan Chua[5], Chin Yen Tay[5], Fanny Thirriot[5], Mun Fai Loke[1], Khean Lee Goh[6], Barry J. Marshall[5], Richard J. Roberts[2], Jamuna Vadivelu[1*]

[1] Department of Medical Microbiology, Faculty of Medicine, University of Malaya, 50603 Kuala Lumpur, Malaysia
[2] New England Biolabs, 240 County Road, Ipswich, MA 01938, USA
[3] Pacific Biosciences, 1380 Willow Road, Menlo Park, CA 94025, USA
[4] PacBio Singapore, Singapore
[5] Marshall Centre, School of Pathology & Laboratory Medicine, The University of Western Australia, 6009 Perth, Australia
[6] Department of Medicine, Faculty of Medicine, University of Malaya, 50603 Kuala Lumpur, Malaysia

*Corresponding author

Email addresses:
WCL: wooncheng.lee@gmail.com
BPA: anton@neb.com
SW: swang@pacificbiosciences.com
PB: pbaybayan@pacificbiosciences.com
SS: ssingh@pacificbiosciences.com
MA: mashby@pacbiosciences.com

EGC: eng.chua@uwa.edu.au

CYT: alfred.tay@uwa.edu.au

FT: fanny.thirriot@uwa.edu.au

MFL: lokemunfai@um.edu.my

KLG: gohkl@um.edu.my

BJM: barry.marshall@uwa.edu.au

RJR: roberts@neb.com

JV: jamuna@um.edu.my

Abstract

Background: The genome of the human gastric pathogen *Helicobacter pylori* encodes a large number of DNA methyltransferases (MTases), some of which are shared among many strains, and others of which are unique to a given strain. The MTases have potential roles in the survival of the bacterium. In this study, we sequenced a Malaysian *H. pylori* clinical strain, designated UM032, by using a combination of PacBio Single Molecule, Real-Time (SMRT) and Illumina MiSeq next generation sequencing platforms, and used the SMRT data to characterize the set of methylated bases (the methylome).

Results: The N4-methylcytosine and N6-methyladenine modifications detected at single-base resolution using SMRT technology revealed 17 methylated sequence motifs corresponding to one Type I and 16 Type II restriction-modification (R-M) systems. Previously unassigned methylation motifs were now assigned to their respective MTases-coding genes. Furthermore, one gene that appears to be inactive in the *H. pylori* UM032 genome during normal growth was characterized by cloning.

Conclusion: Consistent with previously-studied *H. pylori* strains, we show that strain UM032 contains a relatively large number of R-M systems, including some MTase activities with novel specificities. Additional studies are underway to further elucidating the biological significance of the R-M systems in the physiology and pathogenesis of *H. pylori*.

Background

The Gram-negative spiral-shaped bacterium *Helicobacter pylori* persistently colonizes the human stomach and is often associated with chronic gastritis and peptic ulceration. This bacterium is also implicated in more severe gastric diseases that are regarded as an early risk factor for gastric cancer. *H. pylori* strains are genetically diverse and the specific genotypes are associated with clinical outcomes of infection [1, 2]. Previous analyses of *H. pylori* genomes have revealed the presence of a large number of restriction-modification (R-M) systems in several strains [3–5]. The R-M systems are often on mobile elements or associated with recombination-related genes, and divergent among different species and strains [6]. In addition to phase variation, high mutation rate and homologous recombination [7, 8], the R-M system diversity has been proposed to contribute to the genetic variation of the bacteria [9, 10]. Studies have suggested that R-M systems can act as geomarkers that can allow the discrimination of *H. pylori* populations of different geographical origins, thereby reflecting on human migration patterns [9, 11].

In prokaryotes, a DNA methyltransferase (MTase) is often associated with a restriction endonuclease (REase) and forms a R-M system. R-M systems are traditionally divided into four major Types, numbered I, II, III and IV, on the basis of enzyme subunit composition, cofactor requirements and DNA specificity characteristics [12]. Type I systems are encoded by the *hsdS*, *hsdM*, and *hsdR* genes, whose products form multifunctional protein complexes. The HsdS subunit, which composes of two target recognition domains (TRDs), determines the specificity of DNA sequence recognition for both the methylation (HsdM) and cleavage (HsdR) activities. Methylation occurs within each half-recognition-sequence whereas cleavage occurs at a variable distance from the asymmetric recognition site or at an arrested replication fork [13]. A majority of the *H. pylori* R-M systems are of Type II. In contrast to Type I systems, the Type II R-M systems consist of a MTase and a REase that

have enzymatic activities independent of each other, and which often, but not always, occur on independent polypeptides. When these two activities occur on the same polypeptide, the system is denoted Type IIG. Both DNA methylation and cleavage occur within or close to a defined recognition site. Type III systems have two subunits, which are products of the *mod* and *res* genes. The Mod subunit functions independently in hemi-methylation while both subunits are necessary for DNA cleavage. Specificity is determined by the Mod subunit. The Type IV systems comprise a REase that recognizes and cleaves modified DNA.

DNA methylation is an important epigenetic DNA modification in bacteria. The modified bases include 5-methylcytosine (m5C), N4-methylcytosine (m4C) and N6-methyladenine (m6A) [12]. MTases have a crucial role in bacterial biology because these enzymes affect diverse cellular and developmental processes such as gene expression and regulation, cell cycle regulation, anti-mutagenesis, DNA transposition and genome maintenance [14–17]. *H. pylori* is naturally competent and able to take in DNA from the environment [18] as well as being subject to bacteriophage infection [19, 20]. Thus, the MTases might also serve as part of the defence mechanism that protects the genome integrity of the bacteria against transmissible DNA elements. On the other hand, strain-specific MTases are thought to influence the phenotypic traits or virulence in pathogens, host specificity and adaptability to micro-environment [21, 22].

The study of MTases of *H. pylori* enhances our understanding of the pathogenic mechanisms of this organism. The discovery of *hpyIM*, which encodes a Type II MTase that recognizes CATG, revealed that the MTases may play a role in *H. pylori* physiology beyond the methylation function. The expression of *hpyIM* is growth-phase regulated and required for normal bacterial morphology [23]. It was shown that the deletion of *hpyIM* altered the expression of the stress-responsive *dnaK* operon [24]. A Type II MTase, M.HpyAIV, which recognizes GANTC, has been shown to down-regulate the expression of the *katA* gene that

encodes for the catalase, suggesting its importance in the biology of *H. pylori* [25]. The expression of the *modH* gene, a Type III DNA MTase of *H. pylori* which undergoes rapid on/off switching called phase variation, was shown to regulate two proteins, FlaA and FliK, that have important roles in motility [26]. Collectively, these findings provide impetus for dissecting the roles of the DNA MTases in the cellular processes of *H. pylori*.

The implementation of Single Molecule, Real-Time (SMRT) DNA sequencing has allowed the direct identification of methylated bases in synthetic DNA templates, plasmids and bacterial chromosomes [27–29]. This technology monitors the real-time incorporation of fluorescently-labelled nucleotides onto growing DNA chains by individual polymerase molecules [30]. DNA methylation can be detected because the presence of certain modifications on DNA bases in the template delay the incorporation of the nucleotides by the polymerase in a characteristic manner [31]. For substrates of sufficient complexity such as genomic DNA, MTase motifs can be derived *ab initio* by looking for repeating patterns in sequence windows around each methylated base. Furthermore, the fraction of all instances of each motif that is modified can also be determined.

Recently, Krebes and coworkers used SMRT sequencing to analyse the methylomes of two *H. pylori* strains, 26695 and J99 [32]. Despite several earlier studies of the R-M systems in these strains [33–35], the SMRT-assisted analysis provided significant additional insights, including the characterization of Type I and Type III systems and the novel observation of S subunit switching between Type I systems [32]. In addition, another methylome study of five *H. pylori* strains (P12, F16, F30, F32 and F57) by Furuta and co-workers elucidated the relationships between each TRD sequence in S subunit of Type I systems and the corresponding half-site sequence [36]. Given the large numbers of R-M systems typical of *H. pylori* strains in general, it seemed likely to be fruitful to examine additional strains, particularly those isolated from more geographically diverse locations than

the earlier two. *H. pylori* strain UM032 was isolated from a gastroduodenal ulcer patient presenting for gastroscopy at University of Malaya Medical Centre (UMMC), Kuala Lumpur, Malaysia. It is the parental strain for the mice-adapted isolates, *H. pylori* 298 and *H. pylori* 299, and was sequenced using the PacBio platform as described in the previous study [37]. In the present study, the methylome of *H. pylori* UM032 was characterized using SMRT DNA sequencing and compared to those of several previously characterized *H. pylori* strains [32, 36].

Results

Nucleotide sequence accession number

The first annotated *H. pylori* UM032 genome sequence was deposited in DDBJ/EMBL/GenBank with the accession number CP005490 [37]. Here, an updated version of the genome sequence was reported, where the HGAP assembled sequence was corrected by the mapping of Illumina reads. The version described in this paper is CP005490.3.

Methylome analysis of *H. pylori* UM032

SMRT sequencing offers the potential to study DNA methylation in *H. pylori* at a genome-wide scale. Base modifications of the *H. pylori* UM032 genome were analysed, modified sequence motifs were determined, and the MTase responsible for each motif was deduced through a combination of prediction and characterization of cloned and isolated MTases. A total of 63,299 genomic positions were detected as methylated (m4C or m6A). Seventeen functional MTases were identified, of which 14 could be confidently assigned to their MTase sequence specificities based on formerly reported recognition sequences of highly similar examples [38]. The methylated motif G**A**NNNNNNNTAYG, which was reported in *H. pylori* strain F32, was not assigned to a MTase in *H. pylori* UM032 genome.

The remaining two systems demonstrated novel recognition motifs (GAA**A**G and CY**A**NNNNNNNNTRG), which were not previously described in *H. pylori*. The detected methylation motifs are summarized in Table 1, along with the corresponding MTase-encoding genes. All but one active R-M system was of Type II, with only one Type I R-M system and no Type III R-M systems. The analysed methylome of this isolate was deposited in REBASE [38].

Characterization of DNA MTases with unknown specificities

To identify the MTases that recognize and methylate the three unassigned recognition motifs, candidate MTase genes, and their associated S subunits where necessary, were cloned into pRRS and overexpressed in *Escherichia coli* ER2796. Genomic DNA was then isolated from each recombinant strain and subjected to SMRT sequencing to confirm the enzymatic activity of the MTase candidate and to identify the modified motif. Those MTases that were active either in the *H. pylori* UM032 genome or as clones are shown in Table 1, while all MTases not responsible for any activity in the genome or shown to be inactive as clones are shown in Table S3.

K747_03505. This Type I MTase would require association with an S subunit for activity, and the most likely candidate was encoded by the adjacent ORF (K747_03510). Concomitant overexpression of K747_03505 and K747_03510 revealed methylation of the recognition motif G**A**NNNNNNNTAYG. This MTase was designated as M.HpyUM032XII.

K747_03595. This Type IIG gene belonging to the CjeFIII/Eco57-like MTase family was cloned, and SMRT sequencing of genomic DNA from the recombinant *E. coli* strain revealed hemi-methylation of the target sequence GAA**A**G. This MTase was named HpyUM032XIV.

K747_03825. This is a BcgI-like Type IIG R-M system, comprising two S subunit genes (*S1* and *S2*) and a hybrid gene (*RM*) encoding both MTase and REase domains (Figure 1). The two S subunit genes (K747_11950 and K747_11945) are separated by a homopolymeric G repeat, which may have resulted in a previously intact single S subunit becoming split as a result of a frameshift mutation. When the *RM*, *S1*, and *S2* genes were overexpressed together in *E. coli*, the palindromic motif CY**A**NNNNNNNTRG was found to be methylated just as in the genome. This R-M system was named HpyUM032XIII. Interestingly, when the *S1* and *S2* were artificially fused by "correcting" the frameshift and overexpressed with the *RM*, a change of methylation pattern was observed leading to recognition of CY**A**NNNNNNNTTC. This is a new specificity that was not detected in the methylome of *H. pylori* UM032 during normal growth. It was named as HpyUM032XIII-mut1, indicating its artificially derived sequence (Figure 1). Expressing *S2*, but not *S1*, with the *RM* gene gave no activity. On the basis of these results S1, which only encodes one TRD, must be responsible for recognition of the CY**A** half-site. The second TRD would then recognize the half-site GA**A**. The sequences of HpyUM032XIII and HpyUM032XIII-mut1 were deposited in DDBJ/EMBL/GenBank with the accession number KM875507 and KM875508 respectively.t

K747_04185. This is a putative Type III MTase that showed no activity in either *H. pylori* UM032 or when the *mod* gene was cloned into *E. coli* ER2796. A frameshift mutation was identified in the REase gene upstream of the MTase and this may disrupt the functional expression of the MTase if it is transcribed as an operon. Since the cloned MTase was also inactive, the prolonged absence of expression may have allowed the accumulation of less obvious inactivating mutations in the MTase gene itself.

K747_05620. This ORF shares 92.8% amino acid identity with the functional M.Hpy99XVIII of *H. pylori* J99 that methylates TCNNGA. However, when cloned into *E. coli* it did not confer methylation, nor is it active in the genome, assuming that it would have the same recognition specificity as M.Hpy99XVIII.

K747_08715. This is an orphan Type II MTase located within a putative Type III R-M system (between the REase gene K747_08710 and the corresponding unannotated MTase gene). As the two MTases are located adjacent to each other in the genome, both of the genes were cloned and overexpressed both individually and together in *E. coli*. Nevertheless, the overexpressed gene products showed no methylation activity.

K747_10905. Overexpression of this Type I MTase along with its S subunit (K747_10900) in *E. coli* resulted in adenine methylation of the recognition site CC**A**NNNNNNTC. Despite having a functional MTase, no methylation of this motif was detected in the *H. pylori* UM032 genome which could be due to the frameshift in the upstream REase gene that may have disrupted the transcription of this Type I R-M operon.

Discussion

The complete genome sequence of the Malaysian *H. pylori* clinical strain UM032 was obtained using PacBio sequencer as described in the previous study [37]. However, despite the long read length, error rates of single-molecule reads can be as high as 13% [39, 40]. To address this limitation, the strain was sequenced with Illumina Miseq platform in this study to increase genome coverage thereby improved error-correction in single-molecule sequences.

This study describes a methylome analysis of the Malaysian *H. pylori* clinical strain UM032 using SMRT DNA sequencing technology, which can detect m6A and m4C

methylation with high precision. The kinetic signatures of m5C bases may not have been strong enough to properly study. Nonetheless, because of the relatively high sequence coverage [41], one native m5C methylated motif in the *H. pylori* UM032 genomic DNA was identified, G**C**GC. The specificity of the m5C MTases was predicted based on high similarity with homologous examples in other *H. pylori* strains, and so the GCGC motif has been tentatively assigned to the remaining MTase (Table 1). TET treatment of the DNA and cloning of the m5C MTases may reveal additional m5C modification in this genome.

Seventeen R-M systems were identified, of which 16 are of Type II, which is in agreement with previous findings that *H. pylori* encodes mostly Type II R-M systems [11]. *H. pylori* genomes encode unusually high numbers of R-M systems, in particular the Type II R-M systems are highly diverse between strains. However, it is not clear why *H. pylori* possesses this unique characteristic. Three of the recognition motifs (C**A**TG, TCG**A** and ATTA**A**T) present in *H. pylori* UM032 were also detected in other *H. pylori* strains with characterized methylome shown in Table S2, suggesting that they may be essential for the survival and/or maintenance of the genome integrity of *H. pylori* strains in general. The specificity C**A**TG is shared by a previously characterized MTase, M.HpyI, which associates with the epithelial-responsive REase IceAI [24]. The *hpyIM* gene, which encodes M.HpyI, is highly conserved in the genomes of *H. pylori* clinical strains of different geographical origins [23, 35]. Strain UM032 encodes a putative REase, HpyUM032IP, that is 88% identical to IceAI, and is located adjacent to the MTase responsible for methylation of C**A**TG, suggesting this system may have similar epithelial-responsive properties. Two novel methylation motifs were detected in the current study: 1, GAA**A**G, methylated by a Type IIG R-M system designated HpyUM032XIV and; 2, CY**A**NNNNNNNTRG, methylated by another Type IIG R-M system designated HpyUM032XIII. On the other hand, HpyUM032XII, which

recognizes G**A**NNNNNNNTAYG, was the only active Type I R-M system identified in *H. pylori* UM032 genome.

HpyUM032XIII, which resembles the BcgI system in that it consists of a fused RM protein and a separate S protein, also differs from BcgI in that the genetic system encodes two S genes, each of which is one half of the typical length of such genes. It seemed likely that these "half-genes" resulted from a frameshift that had occurred in an ancestral, full-length S gene. Although such frameshift often abolish activity, the cloned system, including RM, S1 and S2 demonstrated MTase activity recognizing the palindromic site CY**A**NNNNNNNTRG. Identical activity was observed when the S2 subunit was omitted, and no activity was observed when S1 was omitted, suggesting the activity resulted from a complex of RM and S1 alone. Surprisingly, when S1 and S2 were artificially fused, the recognition sequence had changed and was now CY**A**NNNNNNNTTC (Figure 1). These observations indicate that S1.HpyUM032XIII must contain a TRD capable of recognizing the half-site CY**A**. Active BcgI, which also recognizes a palindromic sequence, has a stoichiometry of $[(RM)_2S]_2$ [42], and HpyUM032XIII would have a similar stoichiometry, where S is replaced by S1. S.BcgI and S1.HpyUM032XIII must each recognize only a single half-site and therefore require dimerization for functionality. By fusing S1 and S2 into a single protein, two TRDs would be present, and dimerization of S would no longer be required. HpyUM032XIII-mut1 should exhibit a stoichiometry of $(RM)_2S$. A similar phenomenon has been observed in Type I systems such as M.NgoAV [43] and M.Hpy99XVI [32] , but to our knowledge this is the first example of this phenomenon in the context of a Type IIG systems, in which the MTase and REase activities are fused into a single protein. Further studies are required to verify these hypotheses.

Several MTases exhibited different behaviour in various contexts. There was one MTase (K747_10905) of Type I R-M system that was not functional in the genome of *H.*

pylori UM032, but was shown to be active when cloned and overexpressed in *E. coli*. Similar examples of apparent activation when cloned have been noted previously and presumably reflect some silencing mechanism in the genome [32]. Transcriptional silencing [44, 45] or antisense RNA [46] could have been involved in switching off the genes in *H. pylori*, while the lack of such regulation(s) in *E. coli* would result in the expression of this gene. On the other hand, the Type II MTase, K747_08715, and the MTase of a putative Type III R-M system that located adjacent to K747_08715 were both non-functional. This phenomenon could be explained by Nobusato *et al*. [47]. As the R-M systems are often linked with the mobile genetic elements, K747_08715 could have been inserted to this putative Type III R-M system, resulting in inactivation of both systems. A different MTase, K747_05620, which has strong sequence similarity to M.Hpy99XVIII from *H. pylori* J99, was shown to be inactive in both native and cloned contexts. Pairwise alignment revealed that K747_05620 was missing ten amino acid residues from the C-terminus compared to that of M.Hpy99XVIII, which could be the cause of inactivation of the MTase.

Conclusions

This analysis provides yet another illustration of the variability in methylation patterns and MTases that is a hallmark of *H. pylori* biology. Because of its very restricted habitat, it seems unlikely that the large number of potential R-M systems in *H. pylori* strains is needed to protect against bacteriophages. In looking for alternative functions for this extreme methylation it is tempting to speculate that the MTases are involved in the regulation of gene expression that might facilitate rapid adaptation of *H. pylori* to changes in the host environment and thus successful gastric colonisation. They may also play a pivotal role in maintaining genome and strain identity in this naturally competent organism: since multiple

strains are often present in the same niche, DNA methylation may act to limit recombination between strains and thus preserve diversity.

Methods

Bacterial cultivation and preparation of genomic DNA

H. pylori strain UM032 was inoculated onto non-selective lysed blood agar and incubated for three days in humidified air with 10% CO_2 at 37°C. The genomic DNA was extracted from *H. pylori* UM032 using an RTP® Bacteria DNA Mini Kit (Stratec, Germany).

E. coli strains ER2683 [48] and ER2796 [27] were used as hosts for the preparation of plasmid DNA while *E. coli* ER2796 was used to express MTases. All the *E. coli* strains were cultured aerobically overnight at 37°C on Luria-Bertani (LB) agar or in LB broth supplemented with ampicillin (100 µg/ml) when necessary. Genomic DNA from *E. coli* was purified using phenol:methylene chloride extraction as described [49] and resuspended in TE buffer.

Genomic DNA sequencing

The genome of *H. pylori* UM032 was sequenced using a combination of next-generation sequencing platforms. Genomic DNA sequencing was first performed on the Pacific Biosciences (PacBio) RS instrument (Menlo Park, CA) using 10-kb libraries prepared by the manufacturer's kits with C2 chemistry. *H. pylori* UM032 was sequenced on eight SMRT Cells, with one 120-minute movie per Cell, yielding >300× average genome coverage. To improve the quality of the sequence, the genomic DNA was subjected to additional sequencing on an Illumina MiSeq platform. Preparation of the MiSeq library was performed according to the Nextera XT protocol (Ver. May 2012) using Illumina Nextera XT chemistry

(Illumina, San Diego, CA, USA) as previously described with minor modifications [50]. The final libraries were instead normalized by quantification with bioanalyzer (Agilent) and the concentration was adjusted to 4 nM as required by the MiSeq loading protocol. Libraries were sequenced using MiSeq reagent kit v3 (Illumina Inc., San Diego, CA, USA) for a 300-bp paired-end sequencing run using the MiSeq Personal Sequencer (Illumina Inc., San Diego, CA, USA), yielding 135× genome coverage.

E. coli genomes were sequenced using a PacBio RS II instrument (PacBio, Menlo Park, CA, USA). The genomic DNA was treated for 1-hr at 37˚C with RNase I_f (New England Biolabs, Ipswich, MA, USA), sheared to an average size of 5-kb using g-TUBEs (Covaris Inc., Woburn, MA, USA) and purified using the PowerClean DNA Clean-Up Kit (MoBio Laboratories Inc., Carlsbad, CA, USA). PacBio SMRTbell™ template libraries were prepared according to the manufacturer's instructions. SMRT sequencing was performed using Sequencing Reagent 2.0 with DNA polymerase P4. Typically, samples were sequenced with two SMRT Cells using one 120-min movies per Cells, and this typically resulted in coverage of 70-fold across the ER2796 reference. In some cases where methylation levels were low, additional SMRT Cells were employed.

De novo assembly of the *H. pylori* UM032 genome

The results of both sequencing platforms were used to perform *de novo* assembly. The *de novo* assembly of 10-kb insert reads by PacBio sequencing was conducted using the hierarchical genome assembly process (HGAP) version 2.0 [51]. This resulted in a single, complete contig. The raw reads generated from the Illumina platform were aligned to the *H. pylori* UM032 contig using the Geneious R7 in-house read mapper with medium sensitivity

option [52]. Gene prediction was conducted using the NCBI Prokaryotic Genome Annotation Pipeline (PGAP).

Analysis of methylated bases from SMRT® sequencing data

DNA methylation detection was carried out using the kinetic data collected during the genome sequencing process. Genome-wide detection of base modification and the affected motifs were performed using the “RS_Modification_and_Motif_Analysis.1” protocol from PacBio. Motifs were determined using the default quality value (QV) score of 30. While the coverage levels were high enough to warrant raising the QV threshold to a more stringent level, the lower (default) value was chosen to minimize the false negatives. Despite the low threshold, the mean modification QVs of all of the motifs in Table 1 were between 80 and 350. Furthermore, all of the m4C and m6A motifs identified were methylated in 98-100% of the instances of each motif (Table 1), suggesting that none of these were false positives generated by an inappropriately low threshold.

Identification and assignment of MTase genes

The assembled genome was scanned for homologs of R-M system genes using in-house, BLAST-based software (E-value < 1e-11) to identify putative MTases as previously described [53]. Predicted specificities were assigned to candidate MTases based on specificities of previously characterized homologs. The presence of functional motifs, syntenic information, and known characteristics of different MTase types were also used to support or reject those assignments. As examples of characteristic information, Type III and most Type IIG MTases methylate only one strand of their recognition sequence, whereas Type I systems have bipartite recognition sequences consisting of two “half-sites.” MTase candidates with predicted specificities were matched where possible with observed motifs

found in our motif analyses. If a single candidate MTase existed for an observed motif, then that gene was assumed to be responsible for that particular specificity. If multiple candidates existed for a single motif, no automatic assignment was made. When assigning a novel specificity to a given MTase, the MTase gene sequence was cross-checked against other similar genes in REBASE, and the novel specificity against unassigned SMRT-derived motif data in REBASE. In many cases, the same motif occurred in a different genome with an essentially identical MTase or specificity subunit protein sequence, adding weight to the strength of the assignment. MTase information and sequences were deposited in REBASE (http://rebase.neb.com/rebase/rebase.html) [12].

Cloning and over-expression of MTases

Putative MTase and specificity (S) subunit genes were amplified from *H. pylori* UM032 with Q5® High-Fidelity DNA Polymerase (New England Biolabs, Ipswich, MA, USA) using gene-specific oligonucleotide primers and cloned into PCR-amplified pRRS plasmid vector using the Gibson Assembly® Cloning kit (New England Biolabs, Ipswich, MA, USA). Mutations to correct the frameshift in the S subunit of K747_03825 and silent mutations to stabilize polynucleotide repeat sequences were likewise introduced using Gibson Assembly. For example, in K747_03825, the 12-bp repeat sequence GGGGGGGGGGGG was changed to GGAGGAGGCGG, which simultaneously introduced silent mutations to prevent replication slippage and shortened the length to 11, bringing S2 in frame with S1. The expression of all MTase genes was under the regulation of the same *E. coli* P_{lac} promoter present in the pRRS vector. Primer sequences are shown in Table S1.

Recombinant constructs were used to transform *E. coli* ER2683. Restriction analysis was performed to confirm that the bacterial transformants carried the desired plasmid construct.

The plasmid constructs were then used to transform *E. coli* strain ER2796, which lacks endogenous MTase activity. The genomic DNA of the *E. coli* ER2796 recombinant strain was subjected to SMRT sequencing to determine the resulting methylation pattern. Plasmid sequences were confirmed by re-sequencing the PacBio reads against the plasmid reference.

Competing interests

BPA and RJR work for New England Biolabs, a company that commercializes REases, MTases and other enzymes. SS is a full-time employee at PacBio Singapore whereas SW, PB and MA are full-time employees at Pacific Biosciences, a company that commercializes the SMRT DNA sequencing technology.

Authors' contributions

MFL, KLG, BJM, RJR and JV designed the experiments. SW, PB, SS and MA involved in the PacBio sequencing for *H. pylori* and interpretation of the data. EGC, CYT and FT involved in the Illumina MiSeq for *H. pylori* sequencing and interpretation of the data. MFL analysed and assembled the sequencing data. WCL and BPA performed the experiments involving methylation and analysed the data. WCL, BPA and RJR drafted the manuscript. All authors were involved in revising the manuscript, read and approved the final manuscript.

Acknowledgement

This work was supported by University of Malaya-Ministry of Education (UM-MoE) High Impact Research (HIR) Grant UM.C/625/1/HIR/MoE/CHAN/13/2 (Account No. H-50001-A000032) and the Small Business Innovation Research Program (NIGMS) of the National Institutes of Health under award number R44GM105125 to RJR. The content is solely the responsibility of the authors and does not necessarily represent the official views of the

National Institutes of Health.

References

1. Go M, Graham D: **How does *Helicobacter pylori* cause duodenal ulcer disease: the bug, the host, or both?** *J Gastroenterol Hepatol* 1994, **9**:S8–10.

2. Mukhopadhyay AK, Kersulyte D, Jeong J-Y, Datta S, Ito Y, Chowdhury A, Chowdhury S, Santra A, Bhattacharya SK, Azuma T, Nair GB, Berg DE: **Distinctiveness of genotypes of *Helicobacter pylori* in Calcutta, India**. *J Bacteriol* 2000, **182**:3219–26.

3. Baltrus DA, Amieva MR, Covacci A, Lowe TM, Merrell DS, Ottemann KM, Stein M, Salama NR, Guillemin K: **The complete genome sequence of *Helicobacter pylori* strain G27.** *J Bacteriol* 2009, **191**:447–8.

4. Oh JD, Kling-Bäckhed H, Giannakis M, Xu J, Fulton RS, Fulton LA, Cordum HS, Wang C, Elliott G, Edwards J, Mardis ER, Engstrand LG, Gordon JI: **The complete genome sequence of a chronic atrophic gastritis *Helicobacter pylori* strain: evolution during disease progression.** *PNAS USA* 2006, **103**:9999–10004.

5. Alm RA, Ling LL, Moir DT, King BL, Brown ED, Doig PC, Smith DR, Noonan B, Guild BC, Boudewijn L, Carmel G, Tummino PJ, Caruso A, Uria-nickelsen M, Mills DM, Ives C, Gibson R, Merberg D, Mills SD, Jiang Q, Taylor DE, Vovis GF, Trust TJ: **Genomic-sequence comparison of two unrelated isolates of the human gastric pathogen *Helicobacter pylori*.** *Nature* 1999, **397**:176–80.

6. Furuta Y, Kobayashi I, Diversity I: **DNA methyltransferases suggests epigenetics-driven adaptive evolution**. *Mob Genet Elements* 2012, **2**:292–296.

7. Kennemann L, Didelot X, Aebischer T, Kuhn S, Drescher B, Droege M, Reinhardt R, Correa P, Meyer TF, Josenhans C, Falush D, Suerbaum S: ***Helicobacter pylori* genome evolution during human infection.** *PNAS USA* 2011, **108**:5033–5038.

8. Björkholm B, Sjölund M, Falk PG, Berg OG, Engstrand L, Andersson DI: **Mutation frequency and biological cost of antibiotic resistance in *Helicobacter pylori*.** *PNAS USA* 2001, **98**:14607–12.

9. Vale FF, Mégraud F, Vítor JMB: **Geographic distribution of methyltransferases of *Helicobacter pylori*: evidence of human host population isolation and migration.** *BMC Microbiol* 2009, **9**:193.

10. Kobayashi I: **Behavior of restriction-modification systems as selfish mobile elements and their impact on genome evolution**. *Nucleic Acids Res* 2001, **29**:3742–56.

11. Xu Q, Morgan RD, Roberts RJ, Blaser MJ: **Identification of type II restriction and modification systems in *Helicobacter pylori* reveals their substantial diversity among strains.** *PNAS USA* 2000, **97**:9671–6.

12. Roberts RJ, Belfort M, Bestor T, Bhagwat AS, Bickle TA, Bitinaite J, Blumenthal RM, Degtyarev SK, Dryden DTF, Dybvig K, Firman K, Gromova ES, Gumport RI, Halford SE, Hattman S, Heitman J, Hornby DP, Janulaitis A, Jeltsch A, Josephsen J, Kiss A, Klaenhammer TR, Kobayashi I, Kong H, Krüger DH, Lacks S, Marinus MG, Miyahara M, Morgan RD, Murray NE, et al.: **A nomenclature for restriction enzymes, DNA methyltransferases, homing endonucleases and their genes**. *Nucleic Acids Res* 2003, **31**:1805–12.

13. Loenen WAM, Dryden DTF, Raleigh EA, Wilson GG: **Type I restriction enzymes and their relatives**. *Nucleic Acids Res* 2014, **42**:20–44.

14. Kahramanoglou C, Prieto AI, Khedkar S, Haase B, Gupta A, Benes V, Fraser GM, Luscombe NM, Seshasayee ASN: **Genomics of DNA cytosine methylation in *Escherichia coli* reveals its role in stationary phase transcription.** *Nat Commun* 2012, **3**:886.

15. Wion D, Casadesús J: **N6-methyl-adenine: an epigenetic signal for DNA-protein interactions.** *Nat Rev Microbiol* 2006, **4**:183–92.

16. Ishikawa K, Handa N, Sears L, Raleigh EA, Kobayashi I: **Cleavage of a model DNA replication fork by a methyl-specific endonuclease.** *Nucleic Acids Res* 2011, **39**:5489–98.

17. Donczew R, Zakrzewska-Czerwińska J, Zawilak-Pawlik A: **Beyond DnaA: The role of DNA topology and DNA methylation in bacterial replication initiation**. *J Mol Biol* 2014, **426**:2269–2282.

18. Hofreuter D, Odenbreit S, Haas R: **Natural transformation competence in *Helicobacter pylori* is mediated by the basic components of a type IV secretion system**. *Mol Microbiol* 2001, **41**:379–91.

19. Luo C-H, Chiou P-Y, Yang C-Y, Lin N-T: **Genome, integration, and transduction of a novel temperate phage of *Helicobacter pylori*.** *J Virol* 2012, **86**:8781–92.

20. Uchiyama J, Takeuchi H, Kato S, Gamoh K, Takemura-Uchiyama I, Ujihara T, Daibata M, Matsuzaki S: **Characterization of *Helicobacter pylori* bacteriophage KHP30.** *Appl Environ Microbiol* 2013, **79**:3176–84.

21. Heithoff DM: **An essential role for DNA adenine methylation in bacterial virulence**. *Science* 1999, **284**:967–70.

22. Takata T, Aras R, Tavakoli D, Ando T, Olivares AZ, Blaser MJ: **Phenotypic and genotypic variation in methylases involved in Type II restriction-modification systems in *Helicobacter pylori*.** *Nucleic Acids Res* 2002, **30**:2444–52.

23. Xu Q, Peek RM, Miller GG, Blaser MJ: **The *Helicobacter pylori* genome is modified at CATG by the product of *hpyIM*.** *J Bacteriol* 1997, **179**:6807–15.

24. Donahue JP, Israel DA, Torres VJ, Necheva AS, Miller GG: **Inactivation of a *Helicobacter pylori* DNA methyltransferase alters dnaK operon expression following host-cell adherence.** *FEMS Microbiol Lett* 2002, **208**:295–301.

25. Skoglund A, Björkholm B, Nilsson C, Andersson AF, Jernberg C, Schirwitz K, Enroth C, Krabbe M, Engstrand L: **Functional analysis of the M.HpyAIV DNA methyltransferase of *Helicobacter pylori*.** *J Bacteriol* 2007, **189**:8914–21.

26. Srikhanta YN, Gorrell RJ, Steen JA, Gawthorne JA, Kwok T, Grimmond SM, Robins-Browne RM, Jennings MP: **Phasevarion mediated epigenetic gene regulation in *Helicobacter pylori*.** *PLoS One* 2011, **6**:e27569.

27. Clark TA, Murray IA, Morgan RD, Kislyuk AO, Spittle KE, Boitano M, Fomenkov A, Roberts RJ, Korlach J: **Characterization of DNA methyltransferase specificities using single-molecule, real-time DNA sequencing.** *Nucleic Acids Res* 2012, **40**:e29.

28. Flusberg BA, Webster DR, Lee JH, Travers KJ, Olivares EC, Clark TA, Korlach J, Turner SW: **Direct detection of DNA methylation during single-molecule, real-time sequencing.** *Nat Methods* 2010, **7**:461–5.

29. Fang G, Munera D, Friedman DI, Mandlik A, Chao MC, Banerjee O, Feng Z, Losic B, Mahajan MC, Jabado OJ, Deikus G, Clark TA, Luong K, Murray IA, Davis BM, Keren-Paz A, Chess A, Roberts RJ, Korlach J, Turner SW, Kumar V, Waldor MK, Schadt EE: **Genome-wide mapping of methylated adenine residues in pathogenic *Escherichia coli* using single-molecule real-time sequencing.** *Nat Biotechnol* 2012, **30**:1232–9.

30. Eid J, Fehr A, Gray J, Luong K, Lyle J, Otto G, Peluso P, Rank D, Baybayan P, Bettman B, Bibillo A, Bjornson K, Chaudhuri B, Christians F, Cicero R, Clark S, Dalal R, DeWinter A, Dixon J, Foquet M, Gaertner A, Hardenbol P, Heiner C, Hester K, Holden D, Kearns G, Kong X, Kuse R, Lacroix Y, Lin S, et al.: **Real-time DNA sequencing from single polymerase molecules**. *Science* 2009, **323**:133–38.

31. Schadt EE, Banerjee O, Fang G, Feng Z, Wong WH, Zhang X, Kislyuk A, Clark TA, Luong K, Keren-Paz A, Chess A, Kumar V, Chen-Plotkin A, Sondheimer N, Korlach J, Kasarskis A: **Modeling kinetic rate variation in third generation DNA sequencing data to detect putative modifications to DNA bases.** *Genome Res* 2013, **23**:129–41.

32. Krebes J, Morgan RD, Bunk B, Spröer C, Luong K, Parusel R, Anton BP, König C, Josenhans C, Overmann J, Roberts RJ, Korlach J, Suerbaum S: **The complex methylome of the human gastric pathogen Helicobacter pylori.** *Nucleic Acids Res* 2013:1–18.

33. Kong H, Lin L-F, Porter N, Stickel S, Byrd D, Posfai J, Roberts RJ: **Functional analysis of putative restriction-modification system genes in the *Helicobacter pylori* J99 genome**. *Nucleic Acids Res* 2000, **28**:3216–23.

34. Lin L-F, Posfai J, Roberts RJ, Kong H: **Comparative genomics of the restriction-modification systems in *Helicobacter pylori*.** *PNAS USA* 2001, **98**:2740–5.

35. Vitkute J, Stankevicius K, Tamulaitiene G, Maneliene Z, Timinskas A, Berg DE, Janulaitis A: **Specificities of eleven different DNA methyltransferases of *Helicobacter pylori* strain 26695**. *J Bacteriol* 2001, **183**:443–50.

36. Furuta Y, Namba-Fukuyo H, Shibata TF, Nishiyama T, Shigenobu S, Suzuki Y, Sugano S, Hasebe M, Kobayashi I: **Methylome diversification through changes in DNA methyltransferase sequence specificity.** *PLoS Genet* 2014, **10**:e1004272.

37. Khosravi Y, Rehvathy V, Wee WY, Wang S, Baybayan P, Singh S, Ashby M, Ong J, Amoyo AA, Seow SW, Choo SW, Perkins T, Chua EG, Tay A, Marshall BJ, Loke MF, Goh KL, Pettersson S, Vadivelu J: **Comparing the genomes of *Helicobacter pylori* clinical strain UM032 and mice-adapted derivatives.** *Gut Pathogens* 2013, **5**:25.

38. Roberts RJ, Vincze T, Posfai J, Macelis D: **REBASE--a database for DNA restriction and modification: enzymes, genes and genomes.** *Nucleic Acids Res* 2010, **38**:D234–6.

39. Quail M, Smith ME, Coupland P, Otto TD, Harris SR, Connor TR, Bertoni A, Swerdlow HP, Gu Y: **A tale of three next generation sequencing platforms: comparison of Ion Torrent, Pacific Biosciences and Illumina MiSeq sequencers**. *BMC Genomics* 2012, **13**:1.

40. Koren S, Schatz MC, Walenz BP, Martin J, Howard JT, Ganapathy G, Wang Z, Rasko D a, McCombie WR, Jarvis ED, Phillippy AM: **Hybrid error correction and *de novo* assembly of single-molecule sequencing reads**. *Nat Biotechnol* 2012, **30**:693–700.

41. **Detecting DNA Base Modifications** [www.pacb.com/pdf/TN_Detecting_DNA_Base_Modifications.pdf]

42. Kong H: **Analyzing the functional organization of a novel restriction modification system, the BcgI system.** *J Mol Biol* 1998, **279**:823–832.

43. Adamczyk-Poplawska M, Lower M, Piekarowicz A: **Deletion of one nucleotide within the homonucleotide tract present in the *hsdS* gene alters the DNA sequence specificity of Type I restriction-modification system NgoAV.** *J Bacteriol* 2011, **193**:6750–9.

44. Casadesús J, Low DA: **Programmed heterogeneity: epigenetic mechanisms in bacteria.** *J Biol Chem* 2013, **288**:13929–35.

45. Bikard D, Marraffini LA: **Innate and adaptive immunity in bacteria: mechanisms of programmed genetic variation to fight bacteriophages.** *Curr Opin Immunol* 2012, **24**:15–20.

46. Mruk I, Kobayashi I: **To be or not to be: regulation of restriction-modification systems and other toxin-antitoxin systems.** *Nucleic Acids Res* 2014, **42**:70–86.

47. Nobusato A, Uchiyama I, Ohashi S, Kobayashi I: **Insertion with long target duplication: A mechanism for gene mobility suggested from comparison of two related bacterial genomes**. *Gene* 2000, **259**:99–108.

48. Sibley MH, Raleigh EA: **Cassette-like variation of restriction enzyme genes in *Escherichia coli* C and relatives.** *Nucleic Acids Res* 2004, **32**:522–34.

49. Sambrook J, Russell DW: **Purification of nucleic acids by extraction with phenol:chloroform**. *Cold Spring Harb Protoc* 2006, **2006**:pdb.prot4455.

50. Perkins TT, Tay CY, Thirriot F, Marshall B: **Choosing a benchtop sequencing machine to characterise *Helicobacter pylori* genomes**. *PLoS One* 2013, **8**:e67539.

51. Chin C-S, Alexander DH, Marks P, Klammer AA, Drake J, Heiner C, Clum A, Copeland A, Huddleston J, Eichler EE, Turner SW, Korlach J: **Nonhybrid, finished microbial genome assemblies from long-read SMRT sequencing data.** *Nat Methods* 2013, **10**:563–9.

52. Kearse M, Moir R, Wilson A, Stones-Havas S, Cheung M, Sturrock S, Buxton S, Cooper A, Markowitz S, Duran C, Thierer T, Ashton B, Meintjes P, Drummond A: **Geneious basic: an integrated and extendable desktop software platform for the organization and analysis of sequence data.** *Bioinformatics* 2012, **28**:1647–9.

53. Murray IA, Clark TA, Morgan RD, Boitano M, Anton BP, Luong K, Fomenkov A, Turner SW, Korlach J, Roberts RJ: **The methylomes of six bacteria.** *Nucleic Acids Res* 2012, **40**:11450–62.

Figure 1. Schematic representation of the specificity switching of the Type IIG MTase HpyUM032XIII. (A) The S1 and S2 subunits are separated by a homopolymeric tract of 12 G residues at the location shown by ▼, which appears to create a natural frameshift. (B) Reducing the tract length from 12 to 11 corrects the frameshift at ▼, thereby fusing the S1 and S2 subunits. This ‘corrected’ sequence is denoted by ‘mut’, to create fusion of S1 and S2 subunits. (C–D) Expression of individual S subunits with the RM gene shows that the S1 subunit is active in the absence of S2.

Table 1. Methylated motifs detected for *H. pylori* UM032.

Type of RM system	Motifs[a]	Type of modification	No. detected[d]	No. in genome	% detected	Locus tag	Nomenclature
I	G**A**NNNNNNN<u>T</u>AYG	m6A	653	653	100.00	K747_03505	M.HpyUM032XII
IIP	<u>T</u>CG**A**	m6A	526	526	100.00	K747_09985	M.HpyUM032XVII
IIP	C**A**<u>T</u>G	m6A	14370	14370	100.00	K747_04980	M.HpyUM032I
IIP	A**C**N<u>G</u>T	m4C	1005	1104	91.03	K747_10995	M.HpyUM032II
IIP	G**A**<u>T</u>C	m6A	10172	10172	100.00	K747_09245	M.HpyUM032III
IIP	G**A**N<u>T</u>C	m6A	5388	5388	100.00	K747_12490	M.HpyUM032IV
IIP	**C**CG<u>G</u>	m4C	3396	3416	99.41	K747_10000	M.HpyUM032IX
IIP	<u>T</u>CNG**A**	m6A	2530	2532	99.92	K747_05140	M.HpyUM032V
IIP	A<u>T</u>TA**A**T	m6A	857	874	98.05	K747_10980	M.HpyUM032VII
IIP	<u>T</u>GC**A**	m6A	11260	11270	99.91	K747_12120	M.HpyUM032VIII
IIS	G**A**GG	m6A	4578	4579	99.98	K747_08850	M2.HpyUM032VI
IIS	CC**A**TC	m6A	2255	2255	100.00	K747_03690	M1.HpyUM032X
IIP	G**C**<u>G</u>C[e]	m5C	774	2396	32.30	K747_05430	M.HpyUM032XV
IIG	CY**A**NNNNNNN<u>T</u>RG[b]	m6A	2319	2320	99.96	K747_03825	HpyUM032XIII
IIG	GAA**A**G[b]	m6A	2514	4955	50.74	K747_03595	HpyUM032XIV
IIP	G<u>T</u>NN**A**C	m6A	528	528	100.00	K747_06370	M.HpyUM032XI
IIP	G<u>T</u>**A**C	m6A	174	174	100.00	K747_06575	M.HpyUM032XVIII
I	CC**A**NNNNNN<u>T</u>C[b,c]	m6A	-	-	-	K747_10905	M.HpyUM032XVI

[a]The methylated base within the motif is in bold while the methylated base in the complementary strand is underlined.

[b]Novel recognition sequences.

[c]Activity identified only after cloning. No methylation activity was observed in *H. pylori* UM032.

[d]The total number includes motifs occurring on the "+" and "–" strands.

[e]Low percentage detected, due to m5C modification.

Additional files

Additional file.pdf

Figure S1.

Table S1. Oligonucleotide primers used for *H. pylori* putative MTase expression in *E. coli*.

Table S2. Comparison of methylation patterns among *H. pylori* UM032 and various *H. pylori* strains.

Table S3. Other MTase genes in *H. pylori* UM032 not responsible for observed activities.

HpyUM032XIII (K747_03825/K747_11950/K747_11945)

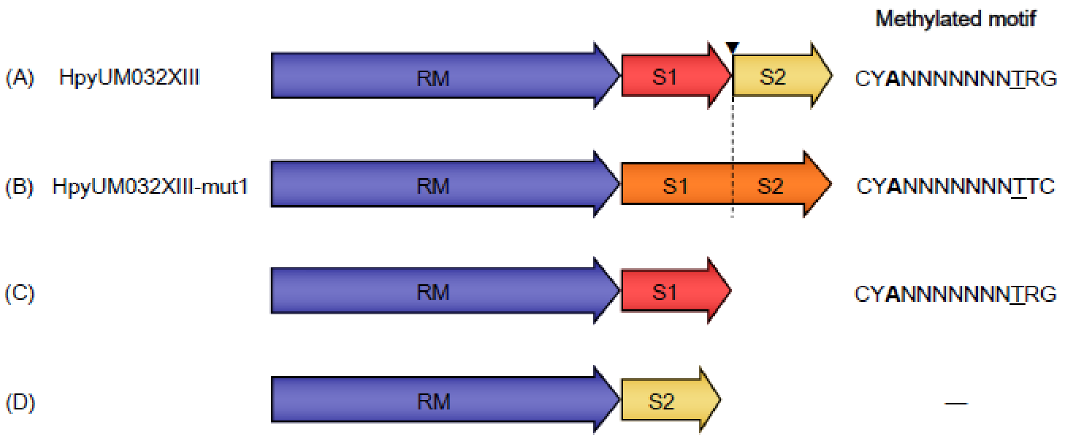

Supplement: Additional file 1: Figure S1. — Modification analysis for H. pylori UM032 at QV 30 and QV 50. Table S1. Oligonucleotide primers used for H. pylori putative MTase expression in E. coli. Table S2. Comparison of methylation patterns among H. pylori UM032 and various H. pylori strains. Table S3. Other MTase genes in H. pylori UM032 not responsible for observed activities. [file 12864_2015_1585_MOESM1_ESM.pdf]
